# Unraveling the Genetic Architecture of Two Complex, Stomata-Related Drought-Responsive Traits by High-Throughput Physiological Phenotyping and GWAS in Cowpea (*Vigna. Unguiculata* L. Walp)

**DOI:** 10.3389/fgene.2021.743758

**Published:** 2021-10-28

**Authors:** Xinyi Wu, Ting Sun, Wenzhao Xu, Yudong Sun, Baogen Wang, Ying Wang, Yanwei Li, Jian Wang, Xiaohua Wu, Zhongfu Lu, Pei Xu, Guojing Li

**Affiliations:** ^1^ Institute of Vegetables, Zhejiang Academy of Agricultural Sciences, Hangzhou, China; ^2^ College of Life Sciences, China Jiliang University, Hangzhou, China; ^3^ Huaiyin Institute of Agricultural Sciences of Xuhuai Region in Jiangsu, Huaian, China; ^4^ State Key Laboratory for Managing Biotic and Chemical Threats to the Quality and Safety of Agro-products, Zhejiang Academy of Agricultural Sciences, Hangzhou, China

**Keywords:** GWAS, phenomic, drought, cowpea, stomatal

## Abstract

Drought is one of the most devasting and frequent abiotic stresses in agriculture. While many morphological, biochemical and physiological indicators are being used to quantify plant drought responses, stomatal control, and hence the transpiration and photosynthesis regulation through it, is of particular importance in marking the plant capacity of balancing stress response and yield. Due to the difficulties in simultaneous, large-scale measurement of stomatal traits such as sensitivity and speed of stomatal closure under progressive soil drought, forward genetic mapping of these important behaviors has long been unavailable. The recent emerging phenomic technologies offer solutions to identify the water relations of whole plant and assay the stomatal regulation in a dynamic process at the population level. Here, we report high-throughput physiological phenotyping of water relations of 106 cowpea accessions under progressive drought stress, which, in combination of genome-wide association study (GWAS), enables genetic mapping of the complex, stomata-related drought responsive traits “critical soil water content” (θ_cri_) and “slope of transpiration rate declining” (K_Tr_). The 106 accessions showed large variations in θ_cri_ and K_Tr_, indicating that they had broad spectrum of stomatal control in response to soil water deficit, which may confer them different levels of drought tolerance. Univariate GWAS identified six and fourteen significant SNPs associated with θ_cri_ and K_Tr_, respectively. The detected SNPs distributed in nine chromosomes and accounted for 8.7–21% of the phenotypic variation, suggesting that both stomatal sensitivity to soil drought and the speed of stomatal closure to completion were controlled by multiple genes with moderate effects. Multivariate GWAS detected ten more significant SNPs in addition to confirming eight of the twenty SNPs as detected by univariate GWAS. Integrated, a final set of 30 significant SNPs associated with stomatal closure were reported. Taken together, our work, by combining phenomics and genetics, enables forward genetic mapping of the genetic architecture of stomatal traits related to drought tolerance, which not only provides a basis for molecular breeding of drought resistant cultivars of cowpea, but offers a new methodology to explore the genetic determinants of water budgeting in crops under stressful conditions in the phenomics era.

## Introduction

Water deficiency caused by soil drought is one of the most severe agricultural problems affecting plant growth and crop yield globally (Gupta et al., 2020). To adapt to drought, plants have evolved the ability that enduring drought stress via changes at the morphological, physiological and molecular levels (Basu et al., 2016), which defining as drought resistance. In plant species, drought resistance includes three major strategies that involving several mechanisms, 1) drought escape, refers to a plant complete its life cycle before the onset of drought; 2) drought avoidance, refers to a plant maintain higher tissue water content to avoid tissue damage under water shortage situations; 3) drought tolerance, refers to a plant sustain a certain level of growth with low internal water content. For any given plant species, it is difficult to resolve the role of different mechanisms of drought resistance, because drought resistance is a dynamic process through regulation of thousands of genes and various metabolic pathways. In Arabidopsis, rice, and other plants, many drought-inducible genes with various functions have been identified, involving different molecular responses and gene pathways (Shinozaki and Yamaguchi-Shinozaki, 2007; Park et al., 2015; Wang et al., 2019; Gupta et al., 2020). In the underground plant organs, when the roots sense the changes of soil moisture, some genes like *EXO7OA3*, *PIN4*, *DEEPER ROOTING1* could modulate root architecture patterning and depth to boost water absorption from soil, thereby improving drought tolerance (Uga et al., 2013; Ogura et al., 2019). In the aboveground plant organs, drought signal via *CLE25* peptide could be transmitted through vasculature to the leaves (Takahashi et al., 2018), through modulate stomatal conductance to improve water use efficiency, and then improve drought resistance. Most genes or transcript factor (TF) involved the hormone abscisic acid (ABA) signaling pathway, which could drive or fine-tune the ABA synthesis to change the ABA content in leaves and guard cells, through engineering the stomatal closure to reduce water lost, thereby promoting drought survival. For example, the Harpin-encoding gene *hrf1*, the RING-finger containing E3 ligase *OsSDIR1* modulate stomatal closure and enhance drought tolerance in rice (Gao et al., 2011; Zhang et al., 2011; You et al., 2013), the ABA receptors PYR/PYL/RCAR and SNF1-related protein kinases (SnRK2 kinases) could improve water use efficiency in A. thaliana (Park et al., 2015; Wang et al., 2019). In addition, some ABA-independent genes also could improve drought resistance, such as the tonoplast aquaporin gene *SlTIP2;2* could maintain larger stomatal aperture and higher whole-plant transpiration under drought stress in tomato (Sade et al., 2009).

For crop plants, sustaining its growth and maintaining yield stability by reducing yield loss under drought conditions is essential for future food security. Exploiting drought resistance gene and understanding the response of cellular signaling to water shortage is key for solving these agricultural problems. Until now, using forward genetics methods such as QTL mapping and genome-wide association mapping, a vast number of genes/QTLs controlling drought-related traits of plants have been identified in different crops such as rice, wheat, maize and cowpea (Mao et al., 2015; Oladosu et al., 2019; Sallam et al., 2019; Ravelombola et al., 2021). Due to the complexity of drought response phenotype, scientists often use a specific trait or several component traits, especially easy to investigate and assay, to unravel plant drought resistance, for example, whole-plant wilting, the senescence of unifoliates, stem greenness and the survival rates of seedling under drought, soluble sugar content, abscisic acid, jasmonic acid (JA), salicylic acid (SA) and ethylene concentration (Castro et al., 2008; Iehisa et al., 2014; Mao et al., 2015; Xu et al., 2015). However, these specific traits or indicators could only reflect plant part-responses at a single point in time, such as the end of drought. They cannot exactly reflect plant how to combat drought, especially plant how to conduct stomatal movements to control water loss in leaves during the progressive soil water content decrease.

As the gateway of both H_2_O evaporation and CO_2_ assimilation in the leaf, stomatal closure can prevent the leaves from desiccation at the cost of photosynthesis, growth, and crop productivity (Schulze et al., 2019). Therefore, investigating plants how to regulate stomatal closure to improve water efficiency and drought resistance is the most direct indicator for drought resistance and the best strategy for drought resistance research. Different species or crop genotypes exhibit different sensitivity in stomatal control, conferring them either profligate or conservative water use behaviors that are related to their water budgeting strategies (Sade et al., 2009). Critical soil water content (θ_cri_), defined as the threshold of volumetric soil water content (VWC) at which a plant starts to restrict its transpiration through stomatal control, is an important quantitative indicator of plant responsiveness to soil drought (Jones, 2006). Some plants such as tomato and cowpea show high θ_cri_ (0.4–0.6) under given ambient environmental conditions, meaning that they respond more promptly to soil water decrease to restrict stomatal conductance; some other plants such as pepper and soybean exhibit relatively lower θ_cri_ (0.14–0.25) under the same conditions, reflecting a more lasting active transpiration until the soil drought becomes quite severe (Xu et al., 2015; Halperin et al., 2017; Dalal et al., 2019). Meanwhile, different crop genotypes also display high variation on the speed of stomatal closure (from full openness to complete closure) at the canopy level (Xu et al., 2015; Halperin et al., 2017; Dalal et al., 2019). How fast and how slow on the stomatal closure means plant could maintain parts of photosynthesis and a certain level of growth, which relating to the yield loss. This specific trait, here we called the slope of transpiration rate declining (K_Tr_), could be also as an accompanying parameter of θ_cri_ using for reflecting drought resistance. Clearly, θ_cri_ and K_Tr_ as two environment-related agronomic traits of plants are closely linked to the balance of drought tolerance and yield penalty in plants under natural drought stress. Understanding the genetic architecture of this two key traits and the governing genes is therefore crucial for harnessing favorable stomatal regulation traits in crop improvement and guiding irrigation management. Traditional manual measurement based on portable apparatuses can measure stomatal conductance (Gs) and transpiration rate (Tr) overtime, which, when combined with the recorded dynamic soil water content data, can be used to calculate the θ_cri_ and K_Tr_. However, it is difficult to acquire comparable θ_cri_ and K_Tr_ data for a large number of individuals simultaneously using this method, thus mapping the genes governing this two key traits faces large challenging.

The recent emergence of several high-throughput physiological phenotyping systems opens a new venue for precise measurement of plant water relations at large scale (Li et al., 2021). One of such platforms developed by Halperin et al. (2017), known as Plantarray, combines gravimetric system, soil and atmospheric probes, controller and irrigation valves in a unit, enabling measurement and calculation of plant water relations as well as the soil-atmosphere parameters during the whole plant growth process. Using a linear regression analysis between transpiration rate and VWC during the drought stress phase, θ_cri_ and K_Tr_ can be derived at the population level (Halperin et al., 2017; Dalal et al., 2019). This revolutionary technology has been successfully used in selection of preferred genotypes in crops including tomato, cowpea and pepper (Xu et al., 2015; Halperin et al., 2017; Dalal et al., 2019). Legumes are staple foods and important vegetables for many cultures all over the world; however, these crops are generally vulnerable to drought as they are commonly grown in rainfed regions (Micheletto et al., 2007; Iglesias-García et al., 2015). Cowpea [*Vigna unguiculata* (L.) Walp], native to Africa, is an important meat alternative for the local poor in the tropics and sub-tropics (Singh et al., 1997). Drought responses of cowpea have been extensively studied, which revealed a wide spectrum of natural variation on stomatal sensitivity to soil drought (Verbree et al., 2015; Xu et al., 2015). In the current study, we established the methodology of large-scale phenotyping of whole-plant water relations in cowpea, which, combined with genome-wide association mapping, enabled forward genetic dissection of the complex, difficult-to-score traits θ_cri_ and K_Tr_, for the first time.

## Materials and Methods

### Plant Materials and Growth Conditions

**FIGURE 1:**
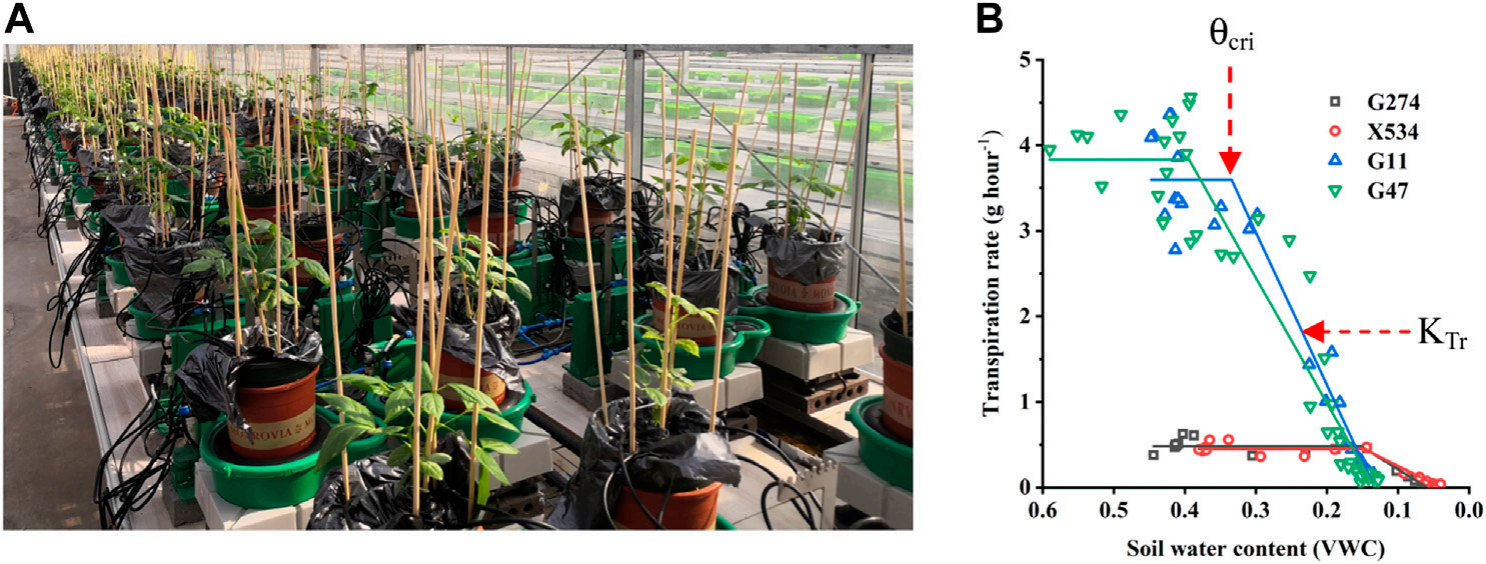
The PlantArray system used in the current study. **(A)** Top view of the automated physiological phenotyping array with 100 measuring units loaded with cowpea seedlings. **(B)** Illustration of the θ _cri_ and K_Tr_ in four representative accessions.

A total of 113 cowpea accessions, which was a subset of the 299-accesion cowpea mini-core previously used in trait-marker association mapping of many traits (Xu et al., 2017), were used in this study (Supplementary Table S1). A lysimetric-based high-throughput physiological phenotyping system, also known as Plantarray (Plant-Ditech, Israel), located in Huai’an (119°01′E, 33°35′N), Jiangsu province, China, was used for physiological phenotyping. This system contains 100 measuring units set up in a semi-controlled greenhouse (Figure 1) with temperature being compensated and ambient light. The experiment design generally followed Xu et al. (2015) and Halperin et al. (2017) with a few modifications. Specifically, 10 seeds from each accession were sown in a tray filled with growing medium (Huaisannong, Huai’an, China) for germination, and three uniform seedlings were transferred to a 3.5 L pot filled with the same medium 2 weeks later. After two more weeks of growth, the pots with plants were transferred to the Plantarray platform, with the pot surface wrapped with plastic film to prevent evaporation. Three replications (pots) were set for each cowpea accession.

### System Setup

Before the pots transferring, weighing lysimeter for each unit was calibrated, and the initial weights for the empty green-bath, soil probes, irrigation drippers, the plastic film for cover and sticks for seedlings bracing were measured. After the pots were put on the green-bath one pot on each unit, the probes, drippers and sticks were added in the pots, and then the experiment was set to run. Because the system could hold a maximum of 100 pots each time, the assay of the 113 accessions were divided into four experiments, spanning from 2018 to 2019 (Table 1). A whole experiment included three stages, normal irrigation, drought stress treatment and water resumption. At the first stage, sufficient irrigation was supplied to ensure the saturation of soil, which occurred in the night during PM 9:00 to AM 3:00, lasting 120–200 s each pulse. At the second stage, irrigation was withheld until the plant’s daily transpiration reached ~10% of that before drought. At the third stage, irrigation was resumed.

**Table 1:** The detailed information for four experiments.

Trial	Accessions	Start time	Normal irrigation	Drought stress	Water recovery	End time
1	73	06/07/2018	06/07–23/07	24/07–03/08	04/08–09/08	09/08/2018
2	30	18/07/2019	18/07–24/07	25/07–06/08	07/08–13/08	13/08/2019
3	31	15/08/2019	15/08–22/08	23/08–03/09	04/09–10/09	10/09/2019
4	9	23/12/2019	23/12–16/01	17/01–13/02	14/02–17/02	17/02/2020

### Raw Data Acquisition and Analysis of θ_cri_ and K_Tr_

The environmental parameters including air temperature, humidity, VPD were recorded by an HC2-S3-L meteo probe (Rotronic, Crawley,United Kingdom) and LI-COR 190 Quantum Sensor (Lincoln, NE, United States). VWC was measured by a soil moisture, salinity and temperature sensor (5TE; Decagon Devices, Pullman, WA, United States). Plant growth and physiological parameters including plant weight, daily transpiration, transpiration rate (Tr) and normalized transpiration (E) were acquired by the Plantarray system automatically as previously detailed in Halperin et al. (2017). The measurement was made every 3 min. The data were simultaneously saved on the online web-based software SPAC analytics (Plant-Ditech, Israel). The average transpiration rate between 11:00 and 13:00 was plotted against VWC for each day of the drought treatment stage. A piecewise linear function was then used to approximatie θ_cri_ and K_Tr_ for each genotype with the following formula: yi = a1+k1*xi, where xi is the θ_cri_, k1 is the K_Tr_ and yi is the max transpiration rate in 1 day, if x < xi, then y = a1+k1*x, else y = yi + k2*(x-xi), and a fitted value *R* ^2^ was used to evaluate the data quality (Halperin et al., 2017). In addition, ANOVA analysis was conducted to investigate the differences among the four experiments using OriginPro 2018.

### Elimination of Batch Effects

In combined data analysis using measurements from different experiments, a linear regression model was used to remove batch effects from the independent experiments (Wheeler and Chambers, 1992). Since VWC and Tr displayed a normal distribution, the specific calculation was performed using the function “lm” in the R software (R3.5 version) where the batch effect was considered as an independent variable. The calculation formula is y = a+x*b, where y is the investigated value, a is the mean value, x is the experiment batch and b is batch effect.

### Population Structure Inference and GWAS

The SNP genotypic data of the 113 cowpea accessions were retrieved from Xu et al. (2017). Population structure was inferred using the software Structure 2.3.4 under the admixture model with a burn-in period of 5,000 followed by 5,000 Markov chain Monte Carlo replications. Five independent runs each were performed with the number of clusters (K) varying from 1 to 10. The optimal K for subgrouping was estimated using STRUCTURE HARVESTER (Earl and vonHoldt, 2012). In addition, an unrooted phylogenetic tree was constructed using Tassel 5.0 under the neighbor-joining method model. Linkage disequilibrium (LD) decay was measured by calculating the square value of correlation coefficient (*r* ^2^) between each SNP pair using Tassel 5.0.

To detect the genomic regions associated with θ_cri_ and K_Tr_, univariate GWAS was conducted on each trait using Tassel 5.0 under the generalized linear model (GLM) with accounting for population structure (Q matrix). The percentage contribution of each SNP to the total phenotypic variation was calculated based on the marker *R* ^2^ values. A multivariate GWAS accounting for population structure and relatedness was also performed on both traits in GEMMA (Zhou and Stephens 2012). For both approaches, only the SNPs showing a minus log_10_-transformed *p* ≥ 2.5 were defined as significant SNPs. If two significant SNPs located in a same LD block, they were considered to represent a same QTL.

### Candidate Genes Analysis

The physical locations of the detected significant SNPs were determined by aligning the marker sequences against the cowpea reference genome V1.1 (Lonardi et al., 2019). Based on the estimated LD decay distance in the genome, the genes residing in the 350 kb upstream and downstream of each SNP locus were retrieved according to the genome annotation. The putative gene functions related to drought stress were analyzed through a literature search using their orthologous genes in Arabidopsis and rice (Shinozaki and Yamaguchi-Shinozaki, 2007; Oladosu et al., 2019). Those having a putative functional relevance to drought response and/or stomatal behavior were considered as candidate causal genes.

## Results

### Phenotypes for Whole-Plant Water Relations in the Single Experiments

**FIGURE 2:**
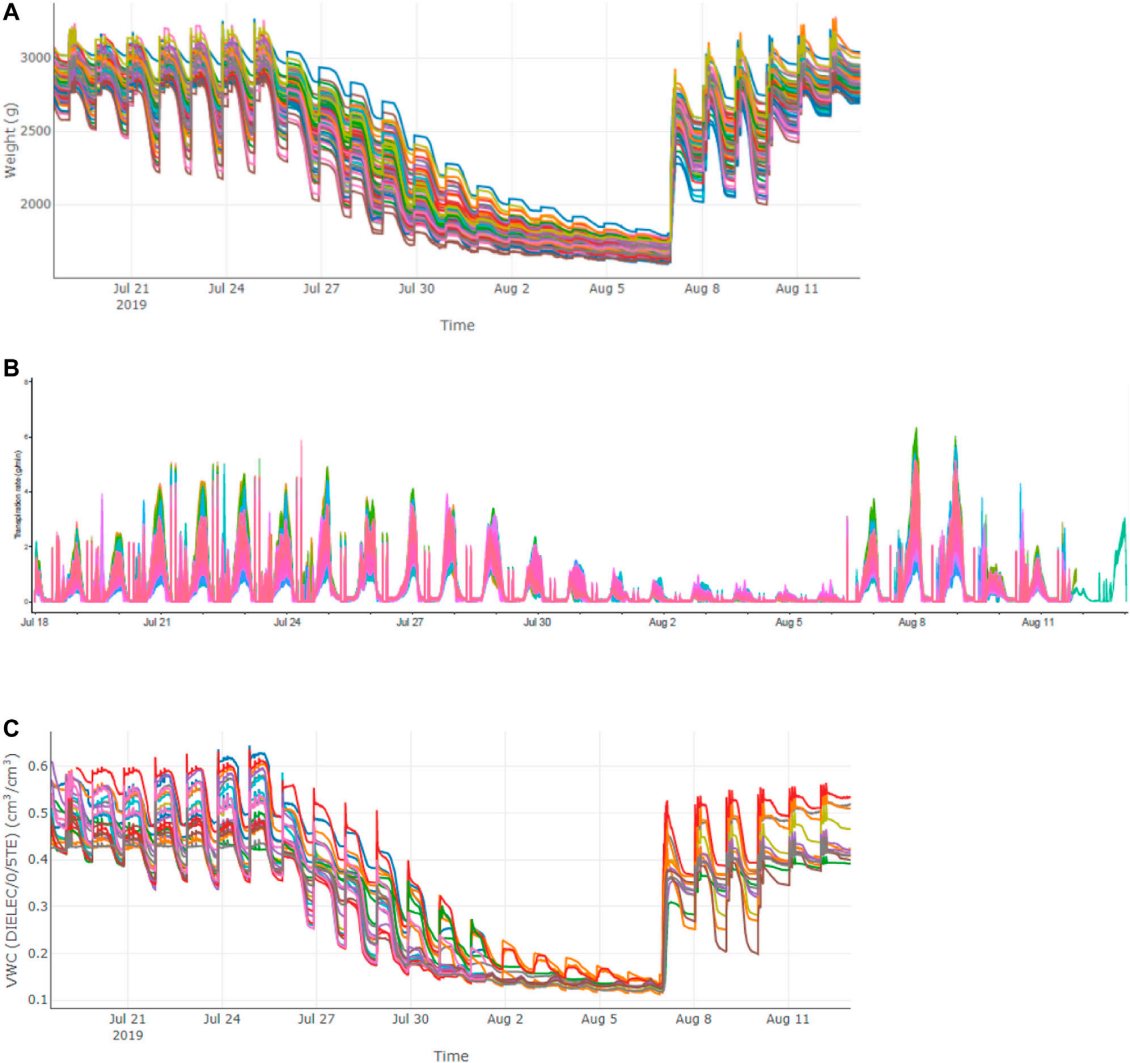
Variations of different parameters in the population in Trial 2 during the course of experiment. **(A)** system weight; **(B)** transpiration rate; **(C)** VWC.

Due to the limited capacity of our system (100 measuring units) and the requirement of biological replicates for physiological assay, the 113 accessions were divided into four batches for phenotyping in two consecutive years and some accessions were tested repeatedly in two trials to reduce experiment error (Table 1). In each experiment, plants were grown under identical ambient environmental condition and subject to the same progressive soil drought treatment imposed by water withholding, which mimicked natural field drought. As shown in Figure 2, the system weight in all experiment showed a similar pattern that increased gradually at the normal irrigation phase, decreased drastically with drought stress treatment and rose back rapidly with water resumption, and ranking from 1,600 to 4,000 g. The average midday transpiration rate (Trm) varied significantly among experiments, largely due to the different experimental seasons, but the CV within each experiment was relatively small, reflecting only the genotypic differences of the trait. Despite varied environmental inputs, Trm of the plants in all experiments remained stable during the initial stage of water withholding, reflecting that all the accessions could maintain the normal transpiration when the soil water is still ample (Figure 2). As the VWC continued to decrease, a reduction of Trm was noted due to the onset of stomata closure (Figure 2). By plotting the dynamic Trm data against the dynamic VWC and using the piecewise linear function, it was clear to see the turning point of Trm (θ_cri_) and the slope of its declining (K_**Tr**_), which provided a physiological measurement of the stomatal sensitivity to gradual soil drought. In Figure 1, the plots of four representative genotypes were shown, from which apparent genotypic variation on θ_cri_ and K_**Tr**_ was observed. Upon re-watering, all plants showed a rapid recovery of Trm (Figure 2).

Using the piecewise linear function, θ_cri_ and K_**Tr**_ for all the genotypes were calculated. Both the θ_cri_ and K_**Tr**_ showed a near-normal distribution except for Trial 4 due to the very small number (9) of accessions included (Supplementary Figure 1). The CV ranged from 0.12 to 0.25 for θ_cri_, and 0.3 to 0.41 for K_**Tr**_, respectively. The broad genotypic variation of these traits indicates that they could be useful in mining the genetic determinants of stomatal sensitivity to soil drought in cowpea. However, the significant between-experiment differences as detected in the four experiments according to ANOVA statistics suggested that batch effects need to be removed before merging the data from individual experiments to create a comprehensive data set for genetic mapping (Table 2; Supplementary Figure 2).

**Table 2:** ANOVA analysis for θ_cri_ and K_Tr_.

Trait	DF	Sum of squares	Mean square	F value	Prob > F
θ_cri_					
Model	3	0.25935	0.08645	30.89261	0
Error	102	0.28544	0.0028		
Total	105	0.54479			
K_Tr_					
Model	3	5735.09872	1911.69957	196.75917	0
Error	102	991.02551	9.71594		
Total	105	6726.12423			

### Data Quality in the Combined Population

**FIGURE 3:**
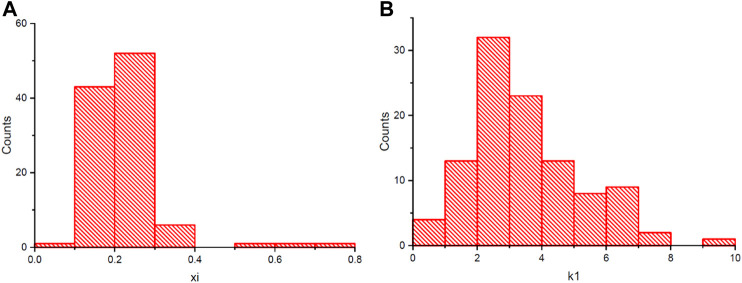
The frequency distribution of θ_cri_ **(A)** and K_Tr_ **(B)** in 106 accessions after removal of batch effects.

To eliminate the batch effects within samples from different experiments, a linear regression model was used to correct the data of the two traits. After deleting data from three accessions with negative value of the fitted *R* ^2^ and four accessions lacking available genotypic data, a final mapping population with a size of 106 accessions was created. As shown in Figure 3, the adjusted θ_cri_ ranged from 0.09 to 0.78 with a median value of 0.21, and the adjusted K_**Tr**_ ranged from 0.44 to 9.17 with a median value of 3.09, after eliminating the batch effects. Both the θ_cri_ and K_**Tr**_ displayed a nearly normal distribution (Figure 3), with the CVs being 0.44 and 0.50, respectively, suggesting that this population is suitable for gene mapping. Approximately 14% of the accessions had a θ_cri_ value smaller than 0.15, and 6.6% of the accessions had a value greater than 0.35, indicating the presence of only a small portion of the genotypes with exceptionally high or low stomatal sensitivity to soil drought. On the contrary, for K_Tr_, 49% of the accessions had a value below the median value of 3.09, suggesting that the majority of the cowpea accessions adjusted their paces of stomatal closure from full openness to complete closure slowly at the canopy scale.

### Genetic Mapping of θ_cri_ and K_Tr_

**FIGURE 4:**
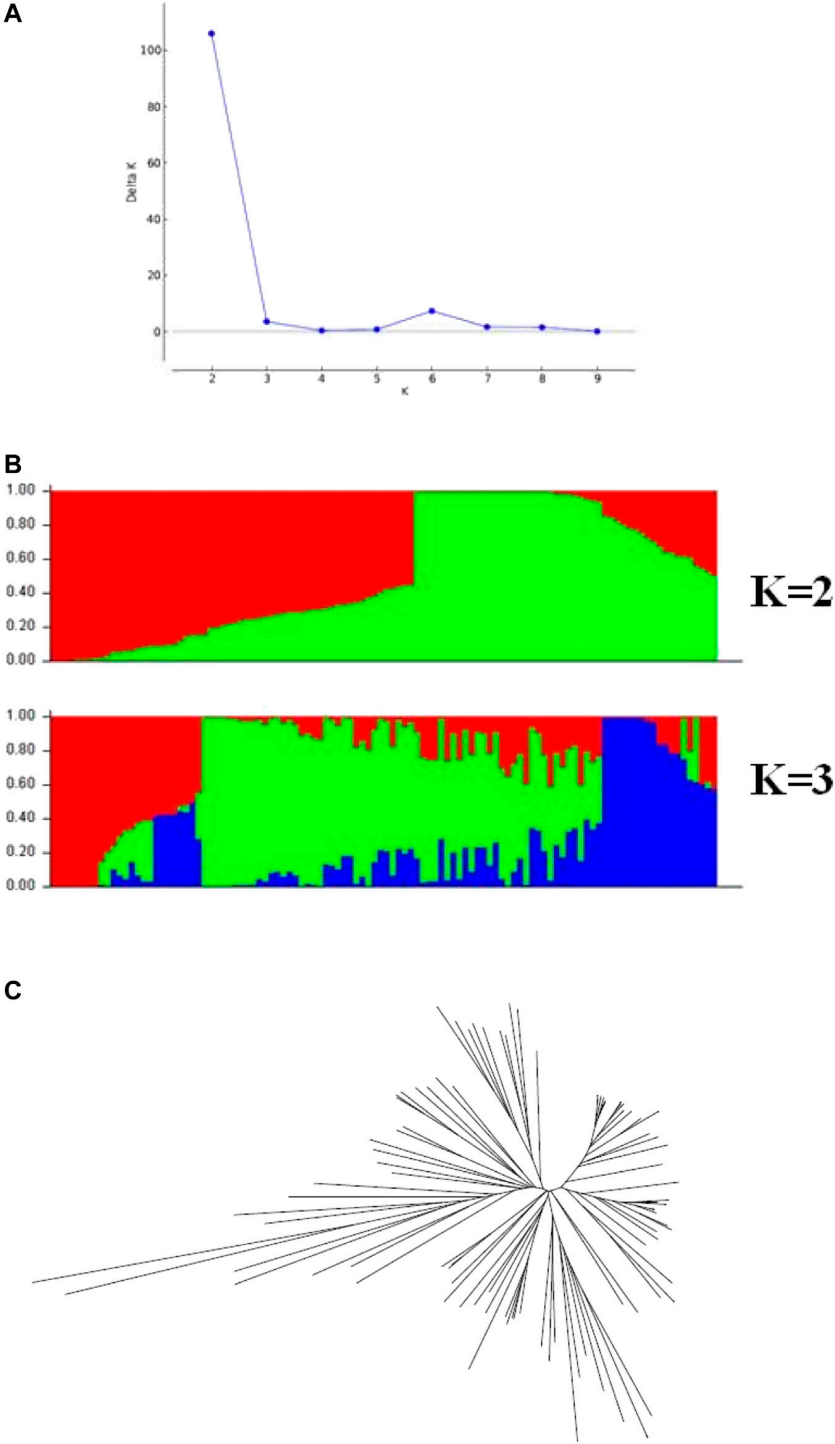
Population structure across the 106 cowpea accessions. **(A)** Delta K values for different numbers of population assumed K values. **(B)** estimated population structure of the germplasm collection inferred at K = 2 and K = 3. **(C)** an unrooted Neighbor-joining tree showing the dendrogram of all samples.

Before performing GWAS of the θcri and K_**Tr**_, the genetic diversity of the 106 accessions was analyzed. Population structure analysis based on the 434 representative SNPs genotype data (Wu et al., 2021) showed that the peak of delta *K* appeared at *K* = 2 (Figure 4), suggesting that the 106-accession subset of the mini-core could be classified into two subpopulations, which is consistent with Xu et al. (2017) from analyzing the entire set of mini-core comprising 299 accessions. The dendrogram of neighbor-joining (NJ) tree also suggested two main branches of these genotypes (Figure 4). Based on their physical location of the SNPs in the reference genome (Lonardi et al., 2019), the LD decays (*r* ^2^ = 0.3) at about 350 kbp across the whole panel (Supplementary Figure 3).

**FIGURE 5:**
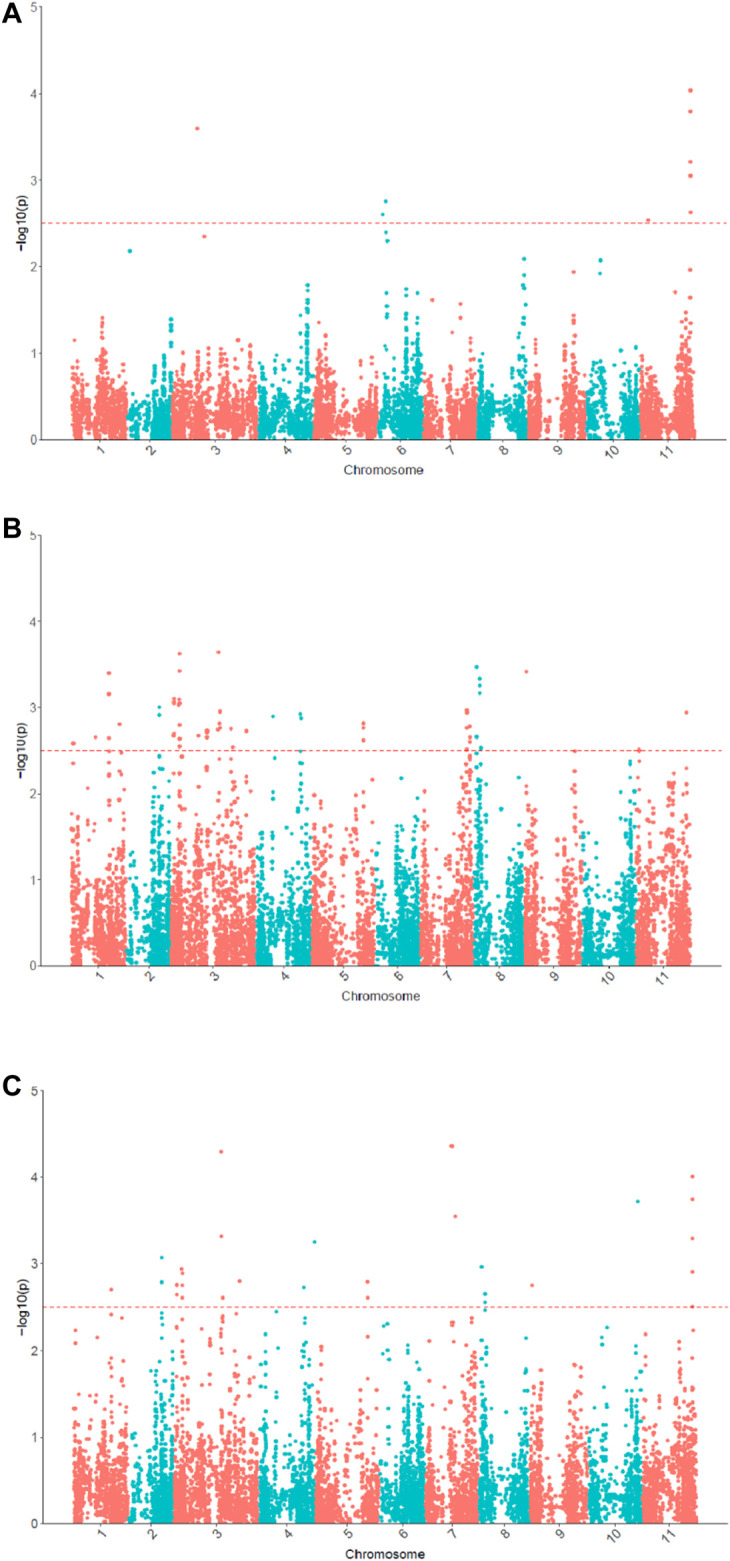
Manhattan plots for θ_cri_ **(A)** and K_Tr_ **(B)** in univariate GWAS and multivariate GWAS for stomatal closure **(C)**.

In the univariate GWAS, 13 significant SNPs associated with θ_cri_ and 64 with K_Tr_ were identified, respectively (Figure 5; Supplementary Table S2). The θ_cri_-associated SNPs were distributed on chromosomes Vu03, Vu06 and Vu11, which accounted for 8.7–21.0% of the phenotypic variation. The K_Tr_-associated SNPs were located on chromosomes Vu01, Vu03, Vu04, Vu05, Vu07, Vu08, Vu09 and Vu11, each explaining 9.1–13.2% of the phenotypic variation. These results indicate that both stomatal sensitivity to soil drought and the speed of stomatal closure to completion in cowpea were controlled by multiple genes with moderate effects. Of the detected SNPs, some formed clusters and located in the same LD blocks, suggesting that they may represent a single locus associated with the trait. For clarity, a final set of six significant SNPs associated with θ_cri_ and fourteen associated with K_Tr_ were reported after retaining only one representative locus in a LD block. There was no overlap between the θ_cri_- and K_Tr_-associated SNPs, indicating that these two traits might be governed by different set of genes.

In the multivariate GWAS, a total of 58 significant SNPs were detected (Figure 5; Supplementary Table S2), which were distributed in all eleven chromosomes except for chromosome Vu05. After merging the SNPs by LD block, 18 representative SNPs were reported in Supplementary Table S2. Of these 18 SNPs, 10 were only detectable in multivariate GWAS, and the remaining eight (or their very closely-neighboring SNPs) viz. 2_53558, 2_31502, 2_07162, 2_12695, 2_06424, 2_15420, 2_03550, 2_16293 were detected also in univariate GWAS. Among these eight SNPs, seven were associated with K_Tr_ and only one was associated with θ_cri_. These results demonstrated that multivariate GWAS could detected more significant SNPs associated with the two traits. By integrating the results of univariate and multivariate GWAS, a final set of 30 SNPs associated with stomatal-closure traits were identified (Supplementary Table S2).

### Candidate Gene Analysis

Given the LD decay distance of 350 kbp in our population, candidate genes in the 350 kb regions up- and down-stream flanking the detected SNPs were searched. This analysis discovered 28 genes as interesting candidate genes (Supplementary Table S3) based on their functional annotations related to drought resistance. These genes included E3 ubiquitin ligase genes, the LEA protein genes, NAC domain proteins, MYB domain protein, amino acid transporters, RING finger domain protein and Zinc finger domain proteins, which were involved in response to drought stress. These results provide a rich candidate gene reservoir for better understanding the mechanisms of stomatal behavior and physiological drought resistance in cowpea, which will help accelerate genetic improvement against drought stress.

## Discussion

Stomatal conductance is known to be a reliable indicator of growth-rate responses to stress (Munns et al., 2010). In response to drought, stomatal closure not only preserves water loss from the plant, but also constrains CO_2_ import to the leaves and thus photosynthesis rate, thereby playing a key role in balancing plant drought resistance and yield (Gupta et al., 2020). Although stomatal control has been extensively studied by physiologists and molecular biologists, the forward genetic dissection of stomatal closure under water stress conditions still largely lagged due to the limited capacity of trait measurements, especially high-throughput measurement at the population level. In the current study, we used a high-throughput physiological phenotyping platform to monitor the dynamic water relations of 106 cowpea accessions simultaneously and continuously. Two specific indicators, θ_cri_ and K_Tr_ were measured and proved to be useful for assessing stomatal responses under progressive water stress conditions. Using phenotypic data of θ_cri_ and K_Tr_, 30 significant SNPs associated with sensitivity or duration of stomatal closure were detected by the GWAS approaches. To our knowledge, this is the first report on genetic mapping of these stomatal closure related traits using forward genetic method in crops.

θ_cri_ and K_Tr_ indicate the critical VWC point at which the stomata start to close and the speed of stomatal closure from full openness to complete closure, respectively. The two traits can, therefore, reflect the property of stomatal response to soil drought from two complementary perspectives. To increase the power of QTL detection, here a combination of univariate GWAS and multivariate GWAS were used. Compared with traditional GWAS or univariate GWAS, multivariate GWAS or multi-trait GWAS showed higher statistical power to detect signals for complex or multiple traits, as has also been demonstrated in studies of the genomic region associated with seed fatty acid in oat and inflorescence and leaf architecture in maize (Carlson et al., 2019; Rice et al., 2020). Our results showed that eight SNPs were detected by both methods, and 10 SNPs were detected only by multivariate GWAS, which proved the greater power of this method and indicate that the 10 SNPs may represent pleiotropic quantitative trait loci for θ_cri_ and K_Tr_.

Due to its high adaptability to drought stress and relatively small genome size (~620 Mb), cowpea has long been used as a model legume crop to understand the genetic basis of drought tolerance in legumes (Ravelombola et al., 2020). In previous studies, based on visually scored morphological traits such as delayed senescence (Dro), stem greenness (Stg), leaf senescence (Scu) and drought tolerance indices, hundreds of significant SNPs associated with drought resistance have been detected by GWAS (Muchero et al., 2013; Xu et al., 2015; Ravelombola et al., 2021). By comparing the locations of these earlier reported SNPs and those mapped in the present study, we found that four known drought-related SNPs/QTLs (Stg 2_25850, Stg 1_0274, Stg 2_07162, Dro-7 1_0067) were co-localized with the newly mapped stomata-associated SNPs, meaning they reside in the same LD decay blocks (distance less than 350 kb) and may represent the same QTLs. These results also imply that the genes underlying stomatal control may ultimately lead to an effect on the morphological responses of cowpea plants to soil drought.

The phytohormone abscisic acid (ABA) plays a critical role in the regulation of stomatal closure to adjust water loss (Wang et al., 2019). Under drought stress, ABA accumulates rapidly and binds its receptors belonging to the PYR1/PYL/RCAR family, which inhibits downstream protein phosphatases to initiate protective responses such as stomatal closure and gene expression reprogramming (Wang et al., 2019). A series of functional genes or transcription factors such as MYB/MYC, NAC proteins, SnRK2, E3 ubiquitin ligase are known to be involved in the ABA signaling pathway (Gao et al., 2011, Shinozaki and Yamaguchi-Shinozaki, 2007; Wang et al., 2019). In our study, 28 gene were listed as interesting candidate genes in the QTL regions based on their putative functional annotations. Most of these genes were presumably to involve in the ABA signaling pathway, including three MYB domain proteins, one NAC domain protein, two E3 ubiquitin ligase proteins, six zinc finger domain proteins and four ring finger containing proteins (Supplementary Table S3). Under drought conditions, these genes may increase drought resistance by adjusting the stomatal aperture. Therefore, ABA signaling pathway is postulated to be the major pathway regulating the stomatal response under drought stress conditions in cowpea. In addition, three late embryogenesis abundant (LEA) proteins were included in the candidate gene list, which may function as chaperone-like protective molecules to combat cellular damage (Babu et al., 2004; Shinozaki and Yamaguchi-Shinozaki, 2007). To confirm the functional relevance of the candidate genes to stomatal regulation, more future work such as fine mapping and positional cloning of these genes are required.

## Conclusion

In the current study, high-throughput physiological phenotyping was employed to quantify two stomatal-related traits that were traditionally difficult-to-score at the population level. Through combining univariate GWAS and multivariate GWAS, we detected 30 significant SNPs associated with θ_cri_ and K_Tr_. The present study provides a so-far rare case of combining high-throughput physiological phenotyping and genetic mapping for forward genetic mapping of stomatal behaviors. Our results lay a foundation for better understanding the molecular mechanisms of stomatal regulation under drought stress conditions as well as marker-assisted breeding for more balanced drought tolerance and yield under drought scenarios in cowpea.

## Data Availability

The original contributions presented in the study are included in the article/Supplementary Material, further inquiries can be directed to the corresponding authors.

## Supplementary Material

Supplementary Figure S1 The frequency distribution for θ_cri_ **(A)** and K_Tr_ **(B)** in the four experiments.

Supplementary Figure S2 The box charts of θ_cri_ and K_Tr_ in the populations in the four experiments. **(A)** original data for θ_cri_; **(B)** original data for K_Tr_; **(C)** θ_cri_ after removing the batch effects; **(D)** θ_cri_ after removing the batch effects.

Supplementary Figure S3 Decay of linkage disequilibrium (LD) in all samples. The strength of LD was measured by *r* ^2^.

Supplementary Table S1 Germplasm lines used in the current study.

Supplementary Table S2 The detected SNPs associated with θ_cri_ and K_Tr_ in univariate GWAS and multivariate GWAS.

Supplementary Table S3 Twenty-eight putative candidate genes surrounding to the detected SNPs.
