# Supplementary figures and images for: Unraveling the Genetic Architecture of Two Complex, Stomata-Related Drought-Responsive Traits by High-Throughput Physiological Phenotyping and GWAS in Cowpea (Vigna. Unguiculata L. Walp)
Source: Front Genet. 2021 Oct 28;12:743758. doi: 10.3389/fgene.2021.743758 (PMC8581254; doi:10.3389/fgene.2021.743758)

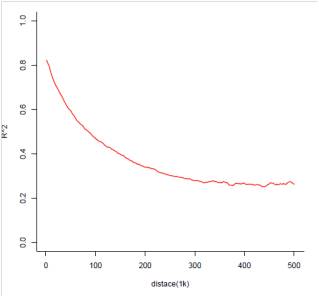

Supplement: Supplementary file 1 [file Image3.JPEG]

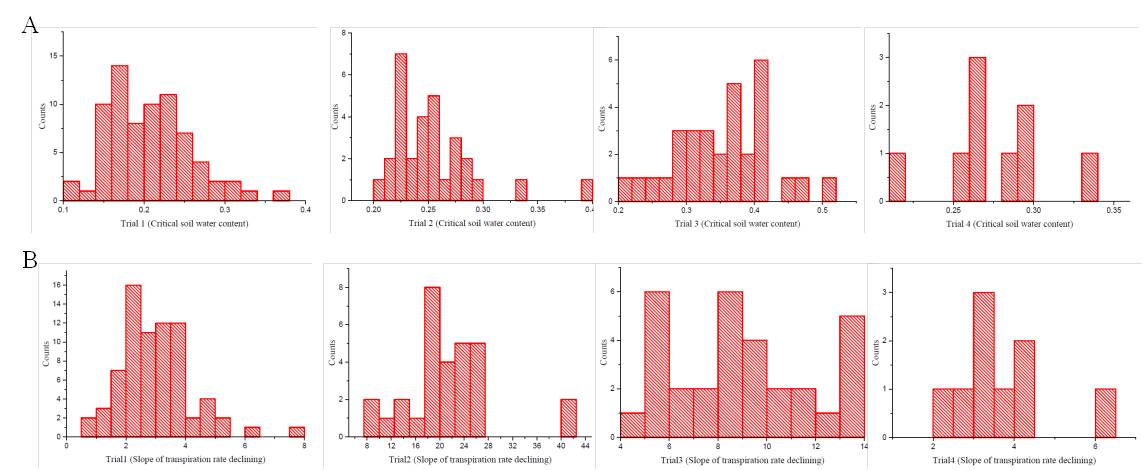

Supplement: Supplementary file 2 [file Image1.JPEG]

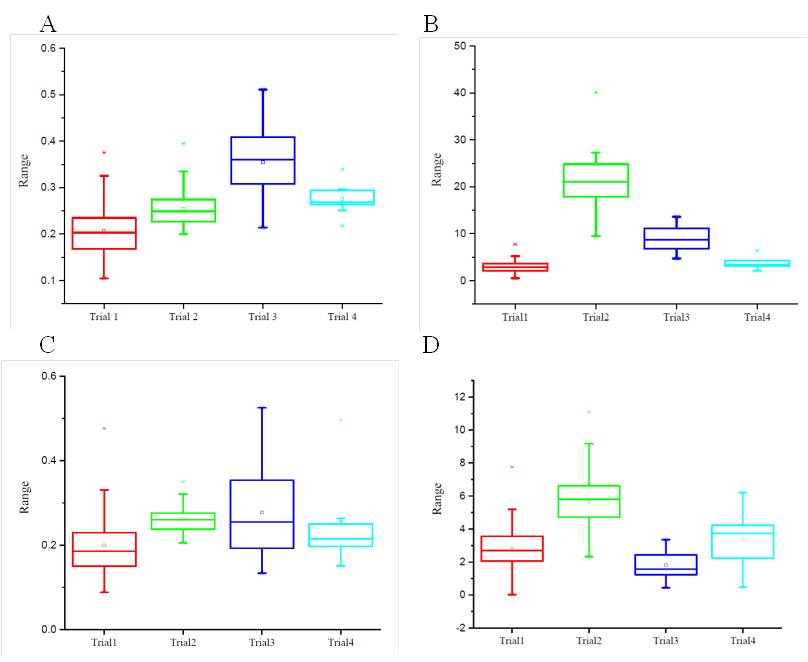

Supplement: Supplementary file 3 [file Image2.JPEG]
