# Supplementary material for: Unraveling the Genetic Architecture of Two Complex, Stomata-Related Drought-Responsive Traits by High-Throughput Physiological Phenotyping and GWAS in Cowpea (Vigna. Unguiculata L. Walp)
Source: Front Genet. 2021 Oct 28;12:743758. doi: 10.3389/fgene.2021.743758 (PMC8581254; doi:10.3389/fgene.2021.743758)
Supplement: Supplementary file 4 [file DataSheet1.docx]

Table S1. Germplasm lines used in the current study.

| **Taxa** | **Name or ID** | **Type** | **Origin** | **Usage type** | **Pod length (cm)** | **Subpopulation assignment** |
| --- | --- | --- | --- | --- | --- | --- |
| G93 | 1162 | Landrace | Southern China | Grain | 34.66 | Subpopulation1 |
| G105 | 1380 | Landrace | Southern China | Grain | 16.42 | Subpopulation1 |
| G42 | 471 | Landrace | Northern China | Grain | 17.68 | Subpopulation1 |
| X461 | Ⅱ7E0266 | Landrace | Northern China | Grain | 28.19 | Subpopulation1 |
| X485 | Ⅱ7E0343 | Landrace | Northern China | Grain | 23.69 | Subpopulation1 |
| X520 | Ⅱ7E0476 | Landrace | Northern China | Grain | 23.07 | Subpopulation1 |
| G47 | 569 | Landrace | Southern China | Grain | 13.87 | Subpopulation1 |
| X540 | Ⅱ7E0760 | Landrace | Northern China | Vegetable | 49.12 | Subpopulation1 |
| X542 | Ⅱ7E0770 | Landrace | Northern China | Grain | 17.25 | Subpopulation1 |
| G22 | 157 | Landrace | Northern China | Grain | 35.05 | Subpopulation1 |
| G23 | 163 | Landrace | Northern China | Grain | 32.59 | Subpopulation1 |
| G34 | 279 | Landrace | Southern China | Vegetable | 54.73 | Subpopulation1 |
| G65 | 873 | Landrace | Northern China | Grain | 27.81 | Subpopulation1 |
| G84 | D1008 | Landrace | Southern China | Grain | 21.17 | Subpopulation1 |
| G323 | CB46 | Cultivar | USA | Grain | 15.26 | Subpopulation1 |
| G342 | UCR5040 | Cultivar | USA | Vegetable | 38.47 | Subpopulation1 |
| G358 | August white | Landrace | Southern China | Grain | 22.84 | Subpopulation1 |
| X3 | Ⅱ7E0008 | Landrace | Northern China | Grain | 31.75 | Subpopulation1 |
| X10 | Ⅱ7E0054 | Landrace | Southern China | Grain | 14.18 | Subpopulation1 |
| X25 | 166 | Landrace | Northern China | Grain | 31.90 | Subpopulation1 |
| X41 | Ⅱ7E0435 | Landrace | Northern China | Grain | 13.08 | Subpopulation1 |
| X72 | D962 | Landrace | Philipine | Grain | 17.01 | Subpopulation1 |
| X103 | D1287 | Landrace | Southern China | Grain | 21.94 | Subpopulation1 |
| TZ30 | Zhijiang282 | Cultivar | Southern China | Vegetable | 54.00 | Subpopulation2 |
| G128 | V07E1401 | Landrace | Southern China | Vegetable | 55.99 | Subpopulation2 |
| G13 | 82 | Landrace | Southern China | Vegetable | 45.30 | Subpopulation2 |
| G158 | Early quanneng | Cultivar | Southern China | Vegetable | 51.90 | Subpopulation2 |
| G189 | Qingjiang80 | Cultivar | Northern China | Vegetable | 52.97 | Subpopulation2 |
| G210 | Guangxibaipi | Cultivar | Southern China | Vegetable | 44.54 | Subpopulation2 |
| G240 | Zhiqing No.3 | Cultivar | Southern China | Vegetable | 50.03 | Subpopulation2 |
| G261 | A3.2.1.1.1.1Q | Improved breeding line | Southern China | Vegetable | 49.71 | Subpopulation2 |
| G264 | Long C△C1.2.1 | Improved breeding line | Southern China | Vegetable | 54.17 | Subpopulation2 |
| G268 | △ClongC5.1.1.5.1Q | Improved breeding line | Southern China | Vegetable | 52.09 | Subpopulation2 |
| G89 | 1128 | Cultivar | Southern China | Vegetable | 41.72 | Subpopulation2 |
| G90 | 1142 | Landrace | Northern China | Vegetable | 49.49 | Subpopulation2 |
| G99 | 1266 | Landrace | Southern China | Vegetable | 55.43 | Subpopulation2 |
| X205 | Youbao | Cultivar | Southern China | Vegetable | 54.01 | Subpopulation2 |
| X451 | Ⅱ7E0241 | Landrace | Southern China | Vegetable | 55.72 | Subpopulation2 |
| X466 | Ⅱ7E0289 | Cultivar | Southern China | Vegetable | 46.66 | Subpopulation2 |
| X535 | Ⅱ7E0711 | Landrace | Southern China | Vegetable | 42.48 | Subpopulation2 |
| G5 | 23 | Cultivar | Southern China | Vegetable | 47.74 | Subpopulation2 |
| X293 | Xinqing | Cultivar | Southern China | Vegetable | 57.59 | Subpopulation2 |
| X372 | Ningbo Lvdai | Cultivar | Southern China | Vegetable | 56.26 | Subpopulation2 |
| X391 | Ⅱ7E0004 | Landrace | Northern China | Vegetable | 51.96 | Subpopulation2 |
| X427 | Ⅱ7E0179 | Landrace | Northern China | Vegetable | 50.77 | Subpopulation2 |
| X503 | Ⅱ7E0411 | Landrace | Northern China | Vegetable | 52.71 | Subpopulation2 |
| X534 | Ⅱ7E0709 | Landrace | Southern China | Vegetable | 36.23 | Subpopulation2 |
| G11 | 62 | Landrace | Southern China | Vegetable | 56.45 | Subpopulation2 |
| G12 | 66 | Landrace | Southern China | Vegetable | 56.40 | Subpopulation2 |
| G24 | 165 | Landrace | Northern China | Vegetable | 62.22 | Subpopulation2 |
| G27 | 197 | Landrace | Southern China | Vegetable | 54.85 | Subpopulation2 |
| G61 | 825 | Landrace | Southern China | Vegetable | 51.23 | Subpopulation2 |
| G73 | 980 | Landrace | Northern China | Vegetable | 45.49 | Subpopulation2 |
| G75 | 983 | Landrace | Northern China | Vegetable | 51.23 | Subpopulation2 |
| G78 | 990 | Landrace | Northern China | Vegetable | 48.94 | Subpopulation2 |
| G115 | VO7E0127 | Landrace | Southern China | Vegetable | 43.32 | Subpopulation2 |
| G230 | Black eyebrow | Cultivar | Northern China | Vegetable | 53.90 | Subpopulation2 |
| G241 | Autumn long bean 512 | Cultivar | Southern China | Vegetable | 35.89 | Subpopulation2 |
| G242 | Purple autumn No.6 | Cultivar | Southern China | Vegetable | 34.14 | Subpopulation2 |
| G249 | Zhijiang109 | Cultivar | Southern China | Vegetable | 48.47 | Subpopulation2 |
| G253 | Zhi 65.5.1.6 | Improved breeding line | Southern China | Vegetable | 50.02 | Subpopulation2 |
| G260 | AQ7 | Improved breeding line | Southern China | Vegetable | 50.31 | Subpopulation2 |
| G262 | AX4.1 | Improved breeding line | Southern China | Vegetable | 45.71 | Subpopulation2 |
| G274 | CAB7.3.1 | Improved breeding line | Southern China | Vegetable | 45.69 | Subpopulation2 |
| G301 | Youlv white bean | Cultivar | Southern China | Vegetable | 47.15 | Subpopulation2 |
| X108 | 1542 | Cultivar | Southern China | Vegetable | 47.26 | Subpopulation2 |
| X126 | VO7E0998 | Landrace | Southern China | Vegetable | 52.73 | Subpopulation2 |
| X153 | Tianxi Yudai | Cultivar | Southern China | Vegetable | 52.23 | Subpopulation2 |
| X208 | Qingbaipi | Landrace | Southern China | Vegetable | 53.28 | Subpopulation2 |
| X32 | Ⅱ7E0270 | Landrace | Southern China | Vegetable | 74.52 | admixed |
| G107 | 1391 | Landrace | Southern China | Vegetable | 49.30 | admixed |
| G19 | 145 | Cultivar | Southern China | Vegetable | 55.93 | admixed |
| X20 | Ⅱ7E0146 | Landrace | Southern China | Vegetable | 43.01 | admixed |
| X445 | Ⅱ7E0222 | Landrace | Southern China | Vegetable | 40.48 | admixed |
| X455 | Ⅱ7E0252 | Landrace | Southern China | Vegetable | 44.68 | admixed |
| X464 | Ⅱ7E0283 | Landrace | Southern China | Vegetable | 52.13 | admixed |
| X476 | Ⅱ7E0308 | Landrace | Southern China | Vegetable | 51.00 | admixed |
| X527 | Ⅱ7E0653 | Landrace | Southern China | Vegetable | 32.30 | admixed |
| X250 | Chunbao | Cultivar | Southern China | Vegetable | 56.48 | admixed |
| X385 | Wujiangdou | Landrace | Southern China | Vegetable | 46.57 | admixed |
| X392 | Ⅱ7E0009 | Landrace | Northern China | Vegetable | 42.54 | admixed |
| X408 | Ⅱ7E0085 | Landrace | Southern China | Vegetable | 64.17 | admixed |
| X414 | Ⅱ7E0104 | Landrace | Southern China | Vegetable | 39.28 | admixed |
| X425 | Ⅱ7E0174 | Landrace | Northern China | Grain | 23.83 | admixed |
| X429 | Ⅱ7E0186 | Landrace | Southern China | Vegetable | 55.07 | admixed |
| X487 | Ⅱ7E0352 | Landrace | Northern China | Vegetable | 37.55 | admixed |
| X530 | Ⅱ7E0668 | Landrace | Southern China | Vegetable | 48.50 | admixed |
| X538 | Ⅱ7E0740 | Landrace | Southern China | Vegetable | 34.77 | admixed |
| G7 | 42 | Landrace | Southern China | Grain | 29.83 | admixed |
| G15 | D97 | Landrace | Philipine | Vegetable | 38.15 | admixed |
| G31 | 253D | Landrace | Southern China | Vegetable | 49.96 | admixed |
| G36 | 353 | Landrace | Northern China | Vegetable | 43.22 | admixed |
| G49 | 587 | Landrace | Southern China | Vegetable | 44.16 | admixed |
| G66 | 879 | Landrace | Northern China | Vegetable | 46.61 | admixed |
| G102 | 1280 | Landrace | Southern China | Vegetable | 31.95 | admixed |
| G312 | Zhijiang dwarf No.1 | Cultivar | Southern China | Grain | 25.55 | admixed |
| G355 | Spotted longbean | Landrace | Southern China | Grain | 32.34 | admixed |
| G356 | Loach longbean | Landrace | Southern China | Vegetable | 52.96 | admixed |
| G360 | 57 | Landrace | Northern China | Vegetable | 67.71 | admixed |
| G362 | 185 | Landrace | Southern China | Vegetable | 39.65 | admixed |
| X6 | D31 | Landrace | Southern China | Vegetable | 40.67 | admixed |
| X33 | Ⅱ7E0278 | Landrace | Southern China | Grain | 25.50 | admixed |
| X51 | D648 | Landrace | Southern China | Vegetable | 38.01 | admixed |
| X64 | 865 | Cultivar | Southern China | Vegetable | 42.23 | admixed |
| X80 | 998 | Landrace | Northern China | Vegetable | 51.24 | admixed |
| X100 | 1271 | Landrace | Southern China | Vegetable | 39.62 | admixed |

| Trait | Marker | CHR | POS | LOD | marker_Rsq |
| --- | --- | --- | --- | --- | --- |
| Ɵcri | 2_46561 | Vu03 | 18913220 | 3.6 | 0.159 |
|  | 2_37707 | Vu06 | 2995298 | 2.6 | 0.087 |
|  | 2_35172 | Vu06 | 5603918 | 2.75 | 0.092 |
|  | 2_40543 | Vu11 | 5665958 | 2.54 | 0.117 |
|  | 2_43920 | Vu11 | 27161530 | 5.13 | 0.21 |
|  | 2_12695 | Vu11 | 38444301 | 4.04 | 0.169 |
| KTr | 2_06426 | Vu01 | 28942082 | 3.43 | 0.112 |
|  | 2_25850 | Vu03 | 6223096 | 3.16 | 0.128 |
|  | 2_17031 | Vu03 | 27786878 | 2.85 | 0.091 |
|  | 2_09217 | Vu03 | 36689824 | 3.17 | 0.129 |
|  | 2_52856 | Vu03 | 58357515 | 2.78 | 0.114 |
|  | 2_05775 | Vu04 | 32579735 | 3 | 0.097 |
|  | 2_55260 | Vu04 | 35055527 | 2.92 | 0.119 |
|  | 2_53558 | Vu05 | 39946763 | 3.11 | 0.101 |
|  | 2_14581 | Vu07 | 38037276 | 2.64 | 0.108 |
|  | 2_31502 | Vu08 | 1472898 | 3.05 | 0.124 |
|  | 2_15451 | Vu08 | 3929804 | 3.2 | 0.13 |
|  | 2_07162 | Vu09 | 1342429 | 3.19 | 0.129 |
|  | 2_01480 | Vu11 | 38882736 | 3.42 | 0.112 |
|  | 2_52525 |  |  | 3.14 | 0.132 |
|  |  |  |  |  |  |
| Multrivariate GWAS | 2_06424 | Vu01 | 28942654 | 2.70 |  |
|  | 1_1472 | Vu02 | 25319809 | 3.07 |  |
|  | 2_14787 | Vu03 | 1818527 | 2.76 |  |
|  | 2_23894 | Vu03 | 5628083 | 2.95 |  |
|  | 2_15420 | Vu03 | 6396423 | 2.89 |  |
|  | 2_03550 | Vu03 | 36461849 | 4.29 |  |
|  | 2_13803 | Vu03 | 37704788 | 2.60 |  |
|  | 2_39815 | Vu03 | 50540693 | 2.80 |  |
|  | 2_26057 | Vu04 | 34164508 | 2.73 |  |
|  | 2_02787 | Vu04 | 42482301 | 3.25 |  |
|  | 2_53558 | Vu05 | 39946763 | 2.80 |  |
|  | 2_22869 | Vu07 | 20111663 | 4.36 |  |
|  | 2_43700 | Vu07 | 22864558 | 3.54 |  |
|  | 2_31502 | Vu08 | 1472898 | 2.97 |  |
|  | 2_16293 | Vu08 | 3958927 | 2.65 |  |
|  | 2_07162 | Vu09 | 1342429 | 2.75 |  |
|  | 2_05168 | Vu10 | 38354471 | 3.72 |  |
|  | 2_12695 | Vu11 | 38444301 | 4.00 |  |

Table S2. The detected SNPs associated with θ_cri_ and K_Tr_ in univariate GWAS and multivariate GWAS.

Table S3. Twenty-eight putative candidate genes surrounding to the detected SNPs.

| Gene ID | Chr | start | end | Annotation information |
| --- | --- | --- | --- | --- |
| Vigun03g070700 | Vu03 | 5836710 | 5838467 | Stress enhanced protein |
| Vigun03g072300 | Vu03 | 5959066 | 5962236 | RING/FYVE/PHD Zinc finger-containing protein |
| Vigun03g072500 | Vu03 | 5972914 | 5975117 | E3 ubiquitin-protein ligase RHA2 |
| Vigun03g075100 | Vu03 | 6245001 | 6246136 | Late embryogenesis abundant (LEA) related genes |
| Vigun03g167500 | Vu03 | 19442656 | 19444901 | HCP-like superfamily protein with MYND-type zinc finger |
| Vigun03g197700 | Vu03 | 28214141 | 28223586 | Zinc finger protein ZPR1 |
| Vigun03g377600 | Vu03 | 58081597 | 58083981 | Ring/U-box superfamily protein |
| Vigun03g377700 | Vu03 | 58099806 | 58102915 | Ring/U-box superfamily protein |
| Vigun04g128100 | Vu04 | 32264081 | 32273724 | DHHC-type zinc finger family protein |
| Vigun04g198800 | Vu04 | 42331393 | 42334999 | NAC domain protein |
| Vigun07g123700 | Vu07 | 22864027 | 22870606 | MYB domain protein |
| Vigun07g124100 | Vu07 | 22946119 | 22948651 | Late embryogenesis abundant (LEA)domain protein |
| Vigun07g261600 | Vu07 | 37774346 | 37777726 | RING/FYVE/PHD Zinc finger-containing protein |
| Vigun07g264500 | Vu07 | 38019033 | 38022903 | Zinc finger CCCH domain protein |
| Vigun07g264800 | Vu07 | 38049087 | 38053045 | Bidirectional amino acid transporter |
| Vigun07g264900 | Vu07 | 38054019 | 38058995 | Bidirectional amino acid transporter |
| Vigun07g265000 | Vu07 | 38063753 | 38067348 | Bidirectional amino acid transporter |
| Vigun09g019400 | Vu09 | 1489262 | 1491034 | Cationic amino acid transporter |
| Vigun09g019600 | Vu09 | 1495016 | 1497064 | Cationic amino acid transporter |
| Vigun10g165300 | Vu10 | 38431144 | 38432590 | MYB domain protein |
| Vigun10g165400 | Vu10 | 38454037 | 38455744 | MYB domain protein |
| Vigun11g041300 | Vu11 | 5934914 | 5937871 | Cationic amino acid transporter |
| Vigun11g091800 | Vu11 | 27317756 | 27318837 | Ring finger containing protein |
| Vigun11g091900 | Vu11 | 27376273 | 27377354 | Ring finger containing protein |
| Vigun11g093000 | Vu11 | 27516998 | 27518169 | Late embryogenesis abundant (LEA)domain protein |
| Vigun11g181500 | Vu11 | 38494713 | 38497647 | B-box zinc finger domain |
| Vigun11g187600 | Vu11 | 38765656 | 38777289 | E3 ubiquitin-protein ligase TRIP12 (TRIP12) |
| Vigun11g188100 | Vu11 | 38815645 | 38818957 | Amino acid permease 3-related |
